# Stone Mountain Medical Association

**Published:** 1887-06

**Authors:** 


### STONE MOUNTAIN MEDICAL ASSOCIATION.

The Stone Mountain Medical Association met at Decatur on the 7th inst., President Word in the Chair.

There was an unusually large attendance. A paper prepared for a previous meeting by Dr. J. H. Green, entitled "A Case of Diphtheria with Rare Complications," came up for consideration. The case was peculiar in that it was attended by a petechial eruption which toward the close of the case covered extensive portions of the surface with dark or ecchymosed patches.

Dr. Hart stated that he had witnessed several cases in which an eruption appeared, and had thought possibly there was a blending of scarlet fever in such cases.

Dr. Mason took position that scarlet fever and diphtheria were identical diseases.

Dr. Goldsmith asked if he had always found a membranous deposit in scarlet fever?

Dr. Mason said not always; yet, had often seen it in the anginose variety.

Dr. Goldsmith regarded scarlet fever and diphtheria as distinct diseases. In Dr. Green's case he thought the petechial eruption indicated that the kidneys were involved.

Dr. Hart said the worst cases of diphtheria had no eruption.

Dr. Word said that the petechial eruption was indicative of alteration of the blood, consequent upon a surcharge of the peculiar poison which gives rise to the malady. He had noticed the same condition in other eruptive diseases. It was well marked in one case of measles which proved fatal. He had seen it in the latter stages of typhoid fever, and in typhus or ship fevers it was a common symptom. He referred to the general tendency to sore throats during the prevalence of diphtheria and scarlet fever by reason of what had been called the "epidemic constitution of the atmosphere."

Dr. Green confirmed this statement, having noticed in one case that an entire family had sore throats wherein a member of the family had scarlet fever. He thought scarlet fever more contagious than diphtheria.

Dr. Goldsmith said he had found scarlet fever more contagious than diphtheria. Diphtheria usually recovered if calomel was given at the outset. He thought little good resulted from local treatment.

Dr. Word said that he treated the disease by both local and constitutional remedies. Of late he had acted in reference to the germ theory. He used calomel at the outset, and gave chlorate of potash internally and turpentine inhalations, and thought well of a mixture of pulverized borax and sulphur frequently blown into the throat through a quill. Tincture of iron and stimulants were used as the more acute stage subsided.

Dr. Hart said that he relied largely upon the internal use of a saturated solution of chlorate of potash, in tablespoonful doses, frequently given. He regards it not a local disease.

Dr. Mason had found good results from the use of a mixture of pulv. chlorate of potash and glycerin. He had seen a mixture of equal parts of oil of eucalyptus and turpentine dissolve rapidly the false membrane, causing it to be easily removed or coughed up.

Dr Jones mentioned a case of abscess in the chest, treated by himself and Dr. Hamilton, in which immense quantities of pus had been discharged. Bichloride solution and carbolic water had been frequently injected and the cavity washed out with good effect. The case is one of much interest and will be reported in full to the Georgia Medical Association.

Dr. Word mentioned a case of epileptiform convulsions of long standing, and almost daily recurrence, that had been arrested for last six weeks by the use of 20 grains of bromide potash and 2 drops tinc. nux vomica three times a day. This treatment was preceded with several small doses of saccharated calomel, which acted freely, producing bilious discharges, and this bilious purging had been once repeated since the case was taken. The case he thought due to reflex irritation from disorder in the alimentary canal.

Dr. Green thought that nearly all cases in young children resulted from worms or other trouble in alimentary canal.

Dr. Goldsmith asked if epileptic paroxysms were attended by a congestive or anæmic state of the brain? He thought there was a determination of blood to the brain, and had recently bled a case with prompt relief. If an anæmic condition existed, bleeding could not relieve and bromide of potash is contra-indicated.

Dr. Mason thought the case mentioned by Dr. Goldsmith was congestive, and not properly to be considered as epileptic.

Dr. Brantly said we should draw the distinction between confirmed and occasional epilepsy and simple convulsions. As a rule, confirmed epileptics were in an anæmic condition.

He asked if the fact had been observed that the red sweet potato, eaten raw, would produce convulsions. In his section so many cases had occurred among the negroes at potato-digging time that there was little doubt that the potatoes caused the trouble, not from mere indigestion as might at first be supposed, but probably from some toxic principle in the skin of the potato. The variety of potato alluded to is commonly called "negro choker." He saw not not less than twenty cases, in all of which the patients had ate the raw potatoes.

He mentioned that he had seen fine results in epilepsy from the use of the sulphate of zinc.

Dr. Brantly closed by asking the opinion of brethren in regard to a case of blindness in a female who had been pregnant and dropsical; had miscarried about five months ago, giving birth to a dead fœtus which had been dead probably ten days at birth and was also dropsical. Patient, though pale and anæmic, has recovered from the dropsical symptoms, but has been blind since miscarriage.

Dr. Word said this was probably the amourosis of albumenuria of which there had been something of late years in the journals. It occurs in the pregnant and in those conditions which lead to puerperal convulsions. The general health should be built up and especial attention given to the kidneys.

The Association adjourned to meet at Decatur the first Thursday in July, at 10 o'clock, a.m., when Dr. Green and Dr. Goldsmith will read papers.

